# Strategies for Regenerating Striatal Neurons in the Adult Brain by Using Endogenous Neural Stem Cells

**DOI:** 10.1155/2011/898012

**Published:** 2011-06-02

**Authors:** Kanako Nakaguchi, Hiroshi Masuda, Naoko Kaneko, Kazunobu Sawamoto

**Affiliations:** Department of Developmental and Regenerative Biology, Nagoya City University Graduate School of Medical Sciences, 1 Kawasumi, Mizuho-cho, Mizuho-ku, Nagoya, Aichi 467-8601, Japan

## Abstract

Currently, there is no effective treatment for the marked neuronal loss caused by neurodegenerative diseases, such as Huntington's disease (HD) or ischemic stroke. However, recent studies have shown that new neurons are continuously generated by endogenous neural stem cells in the subventricular zone (SVZ) of the adult mammalian brain, including the human brain. Because some of these new neurons migrate to the injured striatum and differentiate into mature neurons, such new neurons may be able to replace degenerated neurons and improve or repair neurological deficits. To establish a neuroregenerative therapy using this endogenous system, endogenous regulatory mechanisms that can be co-opted for efficient regenerative interventions must be understood, along with any potential drawbacks. Here, we review current knowledge on the generation of new neurons in the adult brain and discuss their potential for use in replacing striatal neurons lost to neurodegenerative diseases, including HD, and to ischemic stroke.

## 1. Introduction

Huntington's disease (HD) is an adult-onset autosomal-dominant inherited neurodegenerative disorder with progressive symptoms that include involuntary movements, cognitive deficits, and various psychiatric disturbances [1–3]. HD is caused by an expanded CAG repeat in the *huntingtin* gene [4–6]. The expanded CAG repeat gives rise to an abnormally long polyglutamine stretch in the mutant huntingtin, which is toxic to neurons in the striatum and frontal cortex [7]. The most striking pathophysiology of HD is the progressive degeneration of projection neurons and heightened gliosis, leading to a marked atrophy of the striatum and cerebral cortex [8]. So far, although potential therapeutic interventions aimed at suppressing the production of the mutant huntingtin protein and reducing its toxicity have been aggressively pursued [9–13], no effective treatment for HD has been developed. Offering hope, however, are findings by recent studies suggesting that the adult brain's capacity to generate new neurons may be a resource for replacing the affected neurons with newly generated ones. 

 In the mammalian brain, the subventricular zone (SVZ), which is a thin cell layer in the lateral walls of lateral ventricles, continues to produce new neurons during adulthood. Postmortem analyses have shown that the SVZ in HD patients is thickened by increased cell proliferation [14, 15]. In addition, in an HD transgenic mouse model, R6/2 mice carrying the human *HD* gene with long CAG repeats [16] generate new neurons in the SVZ that migrate into the affected striatum and differentiate into mature neurons. Unfortunately, although these alterations may reflect protective responses provoked by the progressive degeneration of striatal neurons owing to HD, they are insufficient to compensate for the pathological process. Nonetheless, these observations may signal the possibility of future interventions that promote the production and migration of new neurons to the damaged striatum and improve the neurological impairments of this disease and/or stop its progression. Here, we review current knowledge on the generation of new neurons (neurogenesis) in the adult brain and discuss its potential for replacing neurons damaged by pathological conditions, including HD.

## 2. Adult Neurogenesis

**Figure 1:**
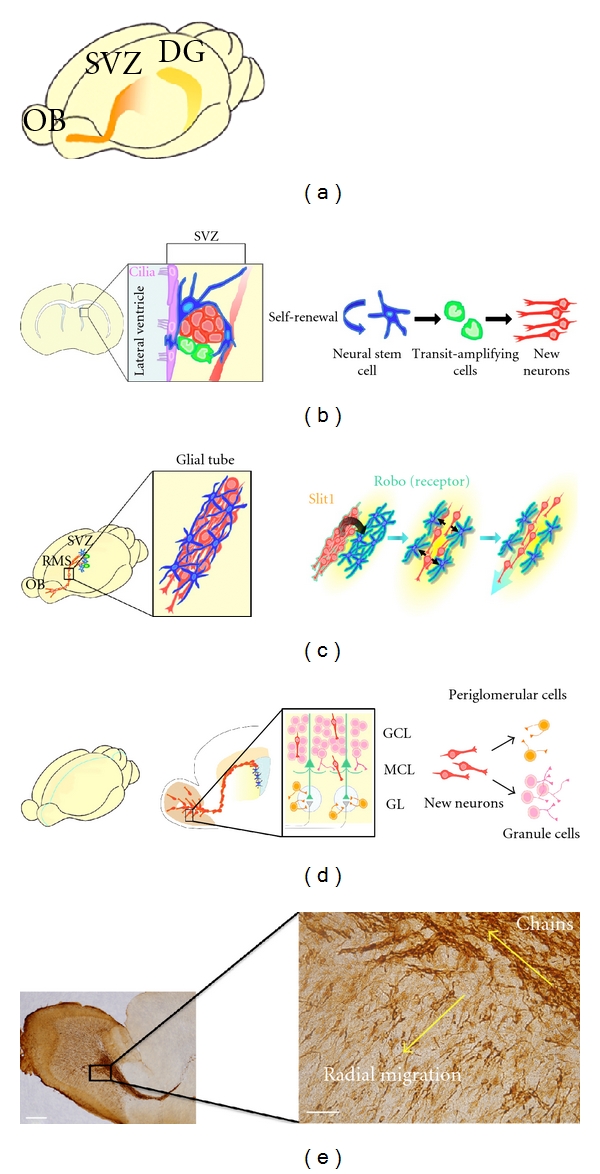
Neurogenesis in the adult brain. (a) Schematic drawing of adult rodent brain showing the two regions, the SVZ and the DG, where new neurons are continuously generated. (b) Location and structure of the SVZ. The SVZ consists of four types of cells, ependymal cells (purple), neural stem cells (blue), transit-amplifying cells (green), and new neurons (red). Neural stem cells directly contact the ventricle with their apical membrane and extend long basal processes that end on blood vessels within the ventricular wall. The neural stem cell proliferates slowly to replicate itself (self-renewal) and to generate transit-amplifying cells. Transit-amplifying cells proliferate quickly, and the progeny differentiate into immature new neurons. (c) Migration of new neurons. New neurons generated in the SVZ migrate into the OB through the RMS, where they form elongated chain-like cell aggregates, which are surrounded by astrocytic tunnels, called glial tubes. The new neurons (red) in the RMS secrete Slit1, whose receptor, Robo, is expressed on astrocytes (blue). Through the Slit1-Robo pathway, the new neurons regulate the morphology of the astrocytes, promoting the formation and maintenance of the glial tubes, which are needed for the neurons' rapid and directional migration. (d) Differentiation of new neurons in the OB. New neurons (red) that reach the OB detach from the chain and migrate radially toward their final destinations, where they differentiate into two types of olfactory interneurons, granule cells (pink) and periglomerular cells (orange) in different layers, the GCL and GL, respectively. (e) Sagittal section of the RMS and OB immunostained for the new neuron marker, DCX. The right panel shows a higher-magnification image of the boxed area at left. Whereas DCX-positive new neurons migrate tangentially in chains, new neurons that migrate radially do so as individuals. Scale bars: left, 500 *μ*m; right, 200 *μ*m. SVZ, subventricular zone; DG, dentate gyrus; OB, olfactory bulb; RMS, rostral migratory stream; GCL, granule cell layer; MCL, mitral cell layer; GL, glomerular layer; DCX, doublecortin.

In the mammalian brain, the production of new neurons in the SVZ and the subgranular zone (SGZ) in the dentate gyrus (DG) of the hippocampus continues during adulthood (Figure 1(a)) [17–21]. Here, we particularly focus on the SVZ because the new neurons generated there have the notable ability to migrate fast and for a long distance in the adult brain. 

There are four types of cells in the adult SVZ: neural stem cells, transit-amplifying cells, newly generated immature neurons, and ependymal cells (Figure 1(b)) [22]. The neural stem cells in the adult SVZ express the astrocyte-specific protein GFAP, and their morphology is not clearly distinguishable from nonneurogenic astrocytes in other brain regions [23, 24]. The SVZ is thought to provide a specific microenvironment, a “stem cell niche,” that supports the neural stem cells' ability to maintain their self-renewing, multipotent state. The process and regulatory mechanisms of neurogenesis in the adult brain have been studied in detail, particularly in rodents. In the SVZ, the stem cells proliferate slowly and continuously to generate transit-amplifying cells, which proliferate quickly; their progeny become immature new neurons. New neurons in the adult rostral migratory stream (RMS), which leads to the olfactory bulb (OB) at the anterior tip of the telencephalon, are still proliferative during their migration. Interestingly, whereas the Wnt-*β*-catenin signaling is involved in the proliferation and differentiation of transit-amplifying cells [25], we found that Diversin, a component of the Wnt pathway, is important in the proliferation of the new neurons [26].

 It is particularly notable that the new neurons migrating through the RMS move quite quickly, 100 *μ*m/h in rodents. This rapid, directional migration is controlled by signals involved in cytoskeletal modification, directional guidance, and interactions between the new neurons and their microenvironment [27–41].

 During their migration in the RMS, the new neurons exhibit a highly polarized morphology with an extended leading and trailing process, and they form elongated cell aggregates called “chains,” within which they move over and past one another (Figure 1(c)). Polysialic acid-neural cell adhesion molecule (PSA-NCAM) and *β*1-integrin expressed on the surface of the new neurons and intracellular Cdk5 signaling are involved in this chain migration [28, 31, 32, 39]. These chains of new neurons move inside tunnels formed by astrocytic processes, termed “glial tubes” (Figure 1(c)) [33, 35, 36], which assist the migration of the new neurons. We recently demonstrated that the relationship between the neurons and glia appears to be interdependent. The tunnel-like arrangement of astrocytes depends on a diffusible protein, Slit1, secreted by the new neurons migrating inside them. (Figure 1(c)) [34]. The neuron-glia interaction may be particularly important for neuronal migration in the adult brain, since it includes a large glial population. In addition, matrix metalloproteases produced by the new neurons, and extracellular matrix molecules including tenascin-C, proteoglycans, and the laminins are all involved in the migration of new neurons in the RMS [28, 33, 37].

 New neurons are guided by extracellular cues to migrate toward the OB, and we found that the directional flow of cerebrospinal fluid (CSF) in the lateral ventricle plays a critical role in their rostral migration [41]. CSF flow is created by the coordinated beating of the multiple ependymal cell cilia, and generates concentration gradients of diffusible proteins, including chemorepellents, secreted into the lateral ventricle. The concentration gradients guide new neurons rostrally toward the OB. In addition, new neurons are guided to the OB by a number of secreted proteins that are produced in the OB, including prokineticin 2 [38], glial cell-line-derived neurotrophic factor (GDNF), and brain-derived neurotrophic factor (BDNF) [29, 40]. 

 New neurons that reach the OB detach from the chain, and the individual cells migrate radially into the granule cell layer and the glomerular layer, where they differentiate into granule cells and periglomerular cells, respectively (Figures 1(d) and 1(e)). The granule cells and periglomerular cells are GABAergic interneurons, which include a small number of periglomerular dopaminergic interneurons. Although the functional significance of these new neurons remains unclear, the neurogenic capacity of the adult rodent brain encourages the hope that this ability might be harnessed to replace neurons destroyed by injury or disease. 

 The human SVZ and DG also retain some ability to generate neurons in adulthood [21, 42], but it is difficult to evaluate human neurogenesis quantitatively, because the experimental procedures are limited. Studies using nonhuman primates and postmortem or surgically dissected human brain tissues indicate that neurogenesis is much less active in the human SVZ than in that of rodents [43–45]. However, new neurons in the human SVZ exhibit a migratory-like polarized morphology and are distributed between the SVZ and OB. These morphological and histological characteristics suggest that new neurons might migrate for long distances in the adult human brain, but this possibility is still controversial [45–49]. In any case, some of the mechanisms that regulate neurogenesis are likely to be common in humans and rodents.

## 3. Alteration of Adult Neurogenesis under Pathological Conditions

Neurogenesis in the adult brain is affected by various brain insults. Following the loss of neurons caused by pathological conditions including stroke and neurodegenerative diseases, newly generated neurons appear in and around the damaged areas. 

**Figure 2:**
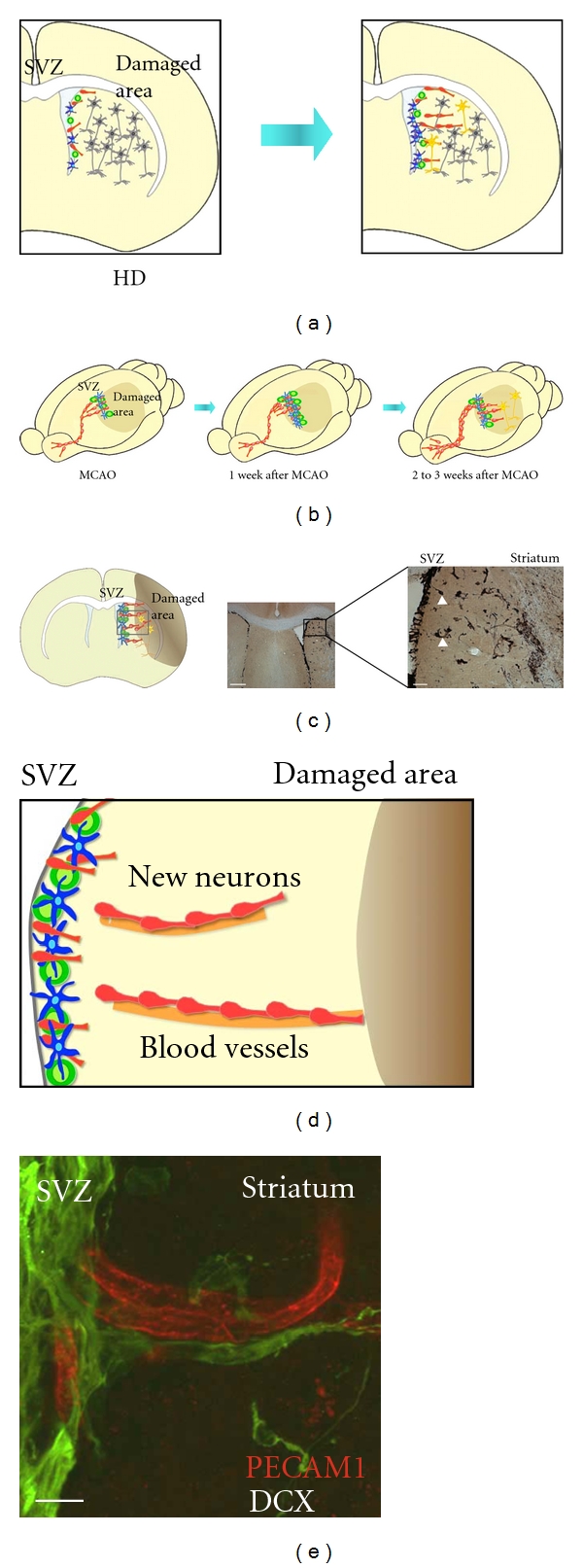
Neurogenesis after neuron loss. (a) Spontaneous neuronal regeneration in HD. In parallel with the progressive degeneration of striatal neurons caused by HD, the production of new neurons in the SVZ increases significantly. These new neurons migrate into the affected striatum, where only some differentiate into mature neurons. (b) Migration of new neurons toward the damaged area in the mouse MCAO model. MCAO causes an infarction in the striatum and adjacent parietal cortex. Within a week of the insult, neural stem cells (blue) and transit-amplifying cells (green) in the SVZ begin to proliferate, and by two or three weeks after MCAO, new neurons (red) migrate and appear at the border of the damaged area in the striatum. (c) At left, a schematic drawing of the coronal brain section 18 days after MCAO. At right a section immunostained for DCX. Boxed area is shown at higher magnification at far right. Many new neurons migrate into the ipsilateral striatum, but not the contralateral striatum. Some new neurons migrate into the striatum in chain-like clusters (far right, arrowheads). Scale bars: left, 500 *μ*m; right, 200 *μ*m. (d) Schematic drawing of new neurons migrating along blood vessels toward the damaged striatum. (e) Confocal projection image of new neurons migrating along blood vessels, in a coronal brain section, 18 days after MCAO. The section was immunostained for DCX (green) and an endothelial cell marker, PECAM-1 (red). Scale bar: 50 *μ*m. MCAO, middle cerebral artery occlusion; SVZ, subventricular zone; OB, olfactory bulb; DCX, doublecortin; PECAM-1, platelet endothelial cell adhesion molecule-1.

 Studies of grade 3 HD patients reported that the SVZ becomes 2.8-fold thicker, with a 2.6-fold increase in the production of new neurons [49, 50]. Although the numbers of transit-amplifying cells and new neurons in the patients' SVZ had increased moderately, the most prominent increase observed was of neural stem cells. In addition, the SVZ of R6/2 mice, a transgenic model for HD, becomes thicker, with a marked increase in the proportion and proliferation of neural stem cells. The self-renewal ability of neural stem cells dissociated from the R6/2 mouse SVZ gradually increases in parallel with disease progression. The rostral migration of new neurons from the SVZ toward the OB is significantly suppressed in these mice; instead, a large population of new neurons migrates laterally into the affected striatum where they differentiate into mature neurons (Figure 2(a)) [16]. The precise mechanism that leads these changes remains to be elucidated; however, the stem cells in the SVZ may function to replace degenerated striatal neurons with new ones, at least in the rodent HD model. In addition, the SVZ-associated neuroregenerative response observed in HD takes place in other pathologies, including ischemic stroke and Parkinson's disease (PD) [35]. 

 Cerebral ischemia is the most commonly studied model of neuronal regeneration after extensive neuronal death (Figure 2(b)). In patients after ischemic stroke, cell proliferation and the production of new neurons in the SVZ are increased, and immature new neurons appear in the cortex close to injured areas and in the striatum close to the SVZ [51–53]. The mechanism and functional significance of the ischemia-induced neurogenesis have mostly been studied using rodent models of transient middle cerebral artery occlusion (MCAO), an experimental model of focal brain ischemia that causes infarction of the ipsilateral striatum and adjacent neocortex [54]. The new neurons generated in the SVZ have a migratory morphology that is directed toward the infarct area, and frequently form chain-like aggregates similar to those observed in the RMS (Figure 2(c)). Our lineage-specific tracing study revealed that the SVZ is almost the sole source of migrating new neurons in the striatum [55]. We also found that new neurons in the striatum are closely associated with astrocytic processes and migrate along blood vessels (Figures 2(d) and 2(e)) [55, 56]. Several proteins produced by the glia and endothelial cells around the infarct area are implicated in this migration, as are their receptors and the MMPs expressed by the new neurons [57–60]. After the migration, most of the new neurons die before they mature, but some survive to differentiate into functional neurons with synaptic contacts. 

 In a rat MCAO model, the number of new striatal neurons increased 31-fold compared with that in sham-operated animals [54]. Using the immunocytochemical detection of BrdU, a thymidine analog that is incorporated into DNA during cell proliferation, and of NeuN, a neuronal marker, newly generated neurons have been identified in the injured striatum of several MCAO models. When BrdU (50 mg/kg) was administered twice a day for 14 days to post-MCAO rats, the density of BrdU/NeuN-colabeled new neurons in the striatum 6 weeks after MCAO was more than 700 cells/mm^3^. On the other hand, in a nonhuman primate (common marmoset) MCAO stroke model, BrdU (50 mg/kg) injections once a day for 18 days after MCAO resulted in a density of BrdU/NeuN-colabeled new striatal neurons 45 days after MCAO that was less than 3 cells/mm^2^, that is, about 50 cells/mm^3^, considering the thickness of the brain sections [61]. These reports suggest that the efficiency of neuronal regeneration is lower in the primate brain, although a precise comparison is not possible due to the difference in BrdU-treatment procedures. Moreover, even in the rat MCAO model, these new neurons could replace only about 0.2% of the dead striatal neurons [54], suggesting that the spontaneous neuronal regeneration would be insufficient to replace the functions of the lost neurons in human patients.

 Because of the low efficiency of neuronal regeneration, interventions that promote this process are now the focus of intense study. Growth factors that promote cell proliferation, differentiation, migration, and/or survival have been reported to act on neural stem cells and/or their progenies to enhance neuronal regeneration [62, 63] (Table 1). In MCAO model animals, epidermal growth factor (EGF) overexpression in the SVZ and the intracisternal administration of fibroblast growth factor 2 (FGF-2) induced increases in the number of proliferating cells in the SVZ [64, 65] whereas infusion of GDNF around the infarct area increased the new neurons in the region by 2-fold [66]. On the other hand, the stromal cell-derived factor-1*α* (SDF-1*α*) CXCR4 signal has been reported to be involved in neuronal migration toward the infarct area [57, 59, 67]. Furthermore, in the R6/2 model mice, BDNF overexpression in the SVZ increased the production of new striatal neurons by about 21-fold, which was enhanced by the co-overexpression of Noggin, a soluble inhibitor of the bone morphogenic proteins (BMPs) [68]. Some of these growth factors may thus be effective in reducing the pathology associated with neuronal loss. 

 Alterations in neurogenesis are also observed in PD, a more common neurodegenerative disease than HD. PD is a motor system disorder characterized by the selective degeneration of dopaminergic neurons in the substantia nigra pars compacta, a basal ganglion of the midbrain, that project into the striatum. Therefore, treatments that increase the dopaminergic stimulation of the striatum improve neurological symptoms. In animal models of PD, after chemically induced dopaminergic denervation of the striatum, new neurons migrate from the SVZ to the striatum, where they differentiate into dopaminergic neurons [78–80]. However, these reports are controversial because other researchers showed that the new neurons did not efficiently migrate into the denervated striatum, but into the OB, and that the SVZ-derived migrating progenitors that did arrive in the striatum never differentiated into mature dopaminergic neurons [81].

 Therefore, even if the SVZ can generate new neurons that migrate to the striatum in pathological conditions, such as HD, these neurons may not replace the functionality of the lost neurons, possibly because they do not mature and differentiate into the needed neuronal types. As mentioned above, new neurons generated in the SVZ become interneurons in the OB. In contrast, more than 90% of striatal neurons are medium-sized spiny projection neurons, and these are the neurons that are mainly injured in HD and cerebral ischemia [82–87]. Although some previous studies reported the regeneration of mature neurons with phenotypes of striatal projection neurons [54, 88], more recent studies demonstrated that, after ischemic stroke, the new neurons that were generated in the SVZ and differentiated in the striatum almost exclusively became calretinin-expressing neurons, a major type of olfactory interneuron [89–91]. These findings suggested that the neurons generated in the SVZ have a limited differentiative capacity for neuronal regeneration. In considering how to attenuate the progression of HD, it is particularly important to learn whether and how we can control the fates of new neurons generated in the adult brain so that they adopt striatal neuronal phenotypes. Further studies are needed to address these points.

## 4. Conclusion

The spontaneous regeneration response of the adult SVZ to pathological neuronal loss does not lead to the regeneration of the lost neurons, because of limitations in the numbers of neurons generated and the fates they adopt. However, many studies support the idea that interventions to increase the production of new neurons in the SVZ and promote their migration, maturation, and survival in the damaged area could be beneficial for treating a variety of neurological deficits, including HD (Table 1). To develop a new therapeutic strategy for pathological neuronal loss, including in cases of HD and stroke, using this system, it will be critical to develop a more precise and comprehensive understanding of the mechanisms that regulate neurogenesis in both physiological and pathological conditions.

## Figures and Tables

**Table 1:** Factors promoting the regeneration of neurons in the striatum. This table lists examples of interventions that could increase the proliferation of neural stem/progenitor cells in the SVZ, enhance the migration of new neurons into the striatum, and promote their differentiation/maturation and survival, which could be promising strategies for replacing degenerated neurons with new ones derived from endogenous neural stem cells of the adult SVZ. SVZ, subventricular zone; RMS, rostral migratory stream; OB, olfactory bulb; TH, tyrosine hydroxylase; EGF, epidermal growth factor; FGF-2, fibroblast growth factor 2; TGF-*α*, transforming growth factor *α*; GDNF, glial cell-derived neurotrophic factor; SDF-1*α*, stromal cell-derived factor 1*α*; BDNF, brain-derived neurotrophic factor; aCSF artificial cerebrospinal fluid; HI, hypoxic-ischemic cerebral injury; MCAO, middle cerebral artery occlusion; 6-OHDA, 6-hydrodopamine; MPTP, 1-methyl-4-phenyl-1,2,3,6-tetrahydroxydropyridine; hNSCs, human neural stem cells; ESCs, embryonic stem cells.

Protein	Model	Technique	Function in neurogenesis	References
EGF	Intact	Infusion into the lateral ventricle using an osmotic pump	Proliferation of SVZ progenitors (18-fold increase compared with saline infusion group)	[69]
	Intact	Infusion into the lateral ventricle using an osmotic pump	Proliferation of SVZ progenitors (9.5-fold increase compared with aCSF infusion group)	[70]
	MCAO	Overexpression in the SVZ	Proliferation of SVZ progenitors (1.7-fold increase compared with control vector treatment group)	[64]

FGF-2	Intact	Infusion into the lateral ventricle using an osmotic pump	Proliferation of SVZ progenitors (2.4-fold increase compared with serum albumin infusion group)	[69]
	Intact	Infusion into the lateral ventricle using an osmotic pump	Proliferation of SVZ progenitors (3.3-fold increase compared with aCSF infusion group)	[70]
	MCAO	Infusion into the cisterna magna	Proliferation of SVZ progenitors (increase compared with vehicle treatment group)	[65]

Angiopoietin 2	6-OHDA	Infusion into the lateral ventricle	Proliferation of SVZ progenitors (increase compared with BSA treatment group)	[71]

TGF-*α*	Intact	Infusion into the lateral ventricle using an osmotic pump	Proliferation of SVZ progenitors (14-fold increase compared with albmin infusion group)	[69]
	6-OHDA	Infusion into the striatum using an osmotic pump	Proliferation of SVZ progenitors (increase compared with PBS infusion group)	[72]
	6-OHDA	Infusion into the striatum using an osmotic pump	Generation of neurons in the striatum (immature neuron, increase compared with aCSF infusion group)	[73]

GDNF	MCAO	Infusion into the striatum using an osmotic pump	Generation of neurons in the striatum (immature neuron, 1.86-fold; mature neuron, 1.2-fold, increase compared with PBS treatment group)	[66]

SDF-1*α*	HI		Migration of implanted hNSCs toward the injured area in ischemic brain slice (increase)	[57]

Tenascin-R	Intact	Tenascin-R expressing cell implantation into the striatum	Migration of SVZ new neurons toward the striatum (4-fold increase compared with the Tenascin nonexpression cell implanted group)	[74]
	Quinolic acid	TNR-expressing ESCs implantation into the striatum	Migration of SVZ new neurons into the striatum (increase compared with TNR-nonexpression ESCs implanted group)	[75]

BDNF	Intact	Infusion into the lateral ventricle using an osmotic pump	Generation of neurons in the OB (increase compared with PBS infusion group)	[76]
	Intact	Infusion into the lateral ventricle using an osmotic pump	Generation of neurons in the striatum (increase compared with PBS infusion group)	[77]
	R6/2	Overexpression in the SVZ	Generation of neurons in the striatum (21-fold increase compared with saline group)	[68]

## References

[1] Bates GP (2005). The molecular genetics of Huntington disease—a history. *Nature Reviews Genetics*.

[2] Montoya A, Price BH, Menear M, Lepage M (2006). Brain imaging and cognitive dysfunctions in Huntington’s disease. *Journal of Psychiatry and Neuroscience*.

[3] Vonsattel JP, Myers RH, Stevens TJ (1985). Neuropathological classification of Huntington’s disease. *Journal of Neuropathology and Experimental Neurology*.

[4] Gusella JF, Wexler NS, Conneally PM (1983). A polymorphic DNA marker genetically linked to Huntington’s disease. *Nature*.

[5] MacDonald ME, Ambrose CM, Duyao MP (1993). A novel gene containing a trinucleotide repeat that is expanded and unstable on Huntington's disease chromosomes. *Cell*.

[6] Snell RG, MacMillan JC, Cheadle JP (1993). Relationship between trinucleotide repeat expansion and phenotypic variation in Huntington’s disease. *Nature Genetics*.

[7] DiFiglia M, Sapp E, Chase KO (1997). Aggregation of huntingtin in neuronal intranuclear inclusions and dystrophic neurites in brain. *Science*.

[8] Ferrante RJ, Kowall NW, Beal MF (1985). Selective sparing of a class of striatal neurons in Huntington’s disease. *Science*.

[9] Bauer PO, Goswami A, Wong HK (2010). Harnessing chaperone-mediated autophagy for the selective degradation of mutant huntingtin protein. *Nature Biotechnology*.

[10] Gil JM, Rego AC (2009). The R6 lines of transgenic mice: a model for screening new therapies for Huntington’s disease. *Brain Research Reviews*.

[11] Gu X, Greiner ER, Mishra R (2009). Serines 13 and 16 are critical determinants of full-length human mutant huntingtin induced disease pathogenesis in HD mice. *Neuron*.

[12] Miller JP, Holcomb J, Al-Ramahi I (2010). Matrix metalloproteinases are modifiers of huntingtin proteolysis and toxicity in Huntington's disease. *Neuron*.

[13] Enokido Y, Tamura T, Ito H (2010). Mutant huntingtin impairs Ku70-mediated DNA repair. *Journal of Cell Biology*.

[14] Curtis MA, Penney EB, Pearson AG (2003). Increased cell proliferation and neurogenesis in the adult human Huntington’s disease brain. *Proceedings of the National Academy of Sciences of the United States of America*.

[15] Curtis MA, Waldvogel HJ, Synek B, Faull RLM (2005). A histochemical and immunohistochemical analysis of the subependymal layer in the normal and Huntington’s disease brain. *Journal of Chemical Neuroanatomy*.

[16] Batista CMC, Kippin TE, Willaime-Morawek S, Shimabukuro MK, Akamatsu W, van der Kooy D (2006). A progressive and cell non-autonomous increase in striatal neural stem cells in the Huntington’s disease R6/2 mouse. *Journal of Neuroscience*.

[17] Altman J (1969). Autoradiographic and histological studies of postnatal neurogenesis. IV. Cell proliferation and migration in the anterior forebrain, with special reference to persisting neurogenesis in the olfactory bulb. *Journal of Comparative Neurology*.

[18] Kaplan MS, Hinds JW (1977). Neurogenesis in the adult rat: electron microscopic analysis of light radioautographs. *Science*.

[19] Cameron HA, Woolley CS, McEwen BS, Gould E (1993). Differentiation of newly born neurons and glia in the dentate gyrus of the adult rat. *Neuroscience*.

[20] Doetsch F, Alvarez-Buylla A (1996). Network of tangential pathways for neuronal migration in adult mammalian brain. *Proceedings of the National Academy of Sciences of the United States of America*.

[21] Eriksson PS, Perfilieva E, Björk-Eriksson T (1998). Neurogenesis in the adult human hippocampus. *Nature Medicine*.

[22] Alvarez-Buylla A, García-Verdugo JM (2002). Neurogenesis in adult subventricular zone. *Journal of Neuroscience*.

[23] Doetsch F, Caille I, Lim DA, Garcia-Verdugo JM, Alvarez-Buylla A (1999). Subventricular zone astrocytes are neural stem cells in the adult mammalian brain. *Cell*.

[24] Garcia ADR, Doan NB, Imura T, Bush TG, Sofroniew MV (2004). GFAP-expressing progenitors are the principal source of constitutive neurogenesis in adult mouse forebrain. *Nature Neuroscience*.

[25] Adachi K, Mirzadeh Z, Sakaguchi M (2007). *β*-catenin signaling promotes proliferation of progenitor cells in the adult mouse subventricular zone. *Stem Cells*.

[26] Ikeda M, Hirota Y, Sakaguchi M (2010). Expression and proliferation-promoting role of diversin in the neuronally committed precursor cells migrating in the adult mouse brain. *Stem Cells*.

[27] Belvindrah R, Hankel S, Walker J, Patton BL, Müller U (2007). *β*1 integrins control the formation of cell chains in the adult rostral migratory stream. *Journal of Neuroscience*.

[28] Bovetti S, Bovolin P, Perroteau I, Puche AC (2007). Subventricular zone-derived neuroblast migration to the olfactory bulb is modulated by matrix remodelling. *European Journal of Neuroscience*.

[29] Chiaramello S, Dalmasso G, Bezin L (2007). BDNF/TrkB interaction regulates migration of SVZ precursor cells via PI3-K and MAP-K signalling pathways. *European Journal of Neuroscience*.

[30] Ghashghaei HT, Weber J, Pevny L (2006). The role of neuregulin-ErbB4 interactions on the proliferation and organization of cells in the subventricular zone. *Proceedings of the National Academy of Sciences of the United States of America*.

[31] Hirota Y, Ohshima T, Kaneko N (2007). Cyclin-dependent kinase 5 is required for control of neuroblast migration in the postnatal subventricular zone. *Journal of Neuroscience*.

[32] Hu H, Tomasiewicz H, Magnuson T, Rutishauser U (1996). The role of polysialic acid in migration of olfactory bulb interneuron precursors in the subventricular zone. *Neuron*.

[33] Jankovski A, Sotelo C (1996). Subventricular zone-olfactory bulb migratory pathway in the adult mouse: cellular composition and specificity as determined by heterochronic and heterotopic transplantation. *Journal of Comparative Neurology*.

[34] Kaneko N, Marín O, Koike M (2010). New neurons clear the path of astrocytic processes for their rapid migration in the adult brain. *Neuron*.

[35] Kaneko N, Sawamoto K (2009). Adult neurogenesis and its alteration under pathological conditions. *Neuroscience Research*.

[36] Lois C, García-Verdugo JM, Alvarez-Buylla A (1996). Chain migration of neuronal precursors. *Science*.

[37] Murase SI, Horwitz AF (2002). Deleted in colorectal carcinoma and differentially expressed integrins mediate the directional migration of neural precursors in the rostral migratory stream. *Journal of Neuroscience*.

[38] Ng KL, Li JD, Cheng MY, Leslie FM, Lee AC, Zhou QY (2005). Neuroscience: dependence of olfactory bulb neurogenesis on prokineticin 2 signaling. *Science*.

[39] Ono K, Tomasiewicz H, Magnuson T, Rutishauser U (1994). N-CAM mutation inhibits tangential neuronal migration and is phenocopied by enzymatic removal of polysialic acid. *Neuron*.

[40] Paratcha G, Ibáñez CF, Ledda F (2006). GDNF is a chemoattractant factor for neuronal precursor cells in the rostral migratory stream. *Molecular and Cellular Neuroscience*.

[41] Sawamoto K, Wichterle H, Gonzalez-Perez O (2006). New neurons follow the flow of cerebrospinal fluid in the adult brain. *Science*.

[42] Bernier PJ, Vinet J, Cossette M, Parent A (2000). Characterization of the subventricular zone of the adult human brain: evidence for the involvement of Bcl-2. *Neuroscience Research*.

[43] Kornack DR, Rakic P (2001). The generation, migration, and differentiation of olfactory neurons in the adult primate brain. *Proceedings of the National Academy of Sciences of the United States of America*.

[44] Sawamoto K, Hirota Y, Alfaro-Cervello C (2011). Cellular composition and organization of the subventricular zone and rostral migratory stream in the adult and neonatal common marmoset brain. *Journal of Comparative Neurology*.

[45] Sanai H, Tramontin AD, Quiñones-Hinojosa A (2004). Unique astrocyte ribbon in adult human brain contains neural stem cells but lacks chain migration. *Nature*.

[46] Bédard A, Parent A (2004). Evidence of newly generated neurons in the human olfactory bulb. *Developmental Brain Research*.

[47] Curtis MA, Kam M, Nannmark U (2007). Human neuroblasts migrate to the olfactory bulb via a lateral ventricular extension. *Science*.

[48] Sanai N, Berger MS, Garcia-Verdugo JM, Alvarez-Buylla A (2007). Comment on “human neuroblasts migrate to the olfactory bulb via a lateral ventricular extension”. *Science*.

[49] Quiñones-Hinojosa A, Sanai N, Soriano-Navarro M (2006). Cellular composition and cytoarchitecture of the adult human subventricular zone: a niche of neural stem cells. *Journal of Comparative Neurology*.

[50] Curtis MA, Penney EB, Pearson J, Dragunow M, Connor B, Faull RLM (2005). The distribution of progenitor cells in the subependymal layer of the lateral ventricle in the normal and Huntington’s disease human brain. *Neuroscience*.

[51] Jin K, Wang X, Xie L (2006). Evidence for stroke-induced neurogenesis in the human brain. *Proceedings of the National Academy of Sciences of the United States of America*.

[52] Macas J, Nern C, Plate KH, Momma S (2006). Increased generation of neuronal progenitors after ischemic injury in the aged adult human forebrain. *Journal of Neuroscience*.

[53] Martí-Fàbregas J, Romaguera-Ros M, Gómez-Pinedo U (2010). Proliferation in the human ipsilateral subventricular zone after ischemic stroke. *Neurology*.

[54] Arvidsson A, Collin T, Kirik D, Kokaia Z, Lindvall O (2002). Neuronal replacement from endogenous precursors in the adult brain after stroke. *Nature Medicine*.

[55] Yamashita T, Ninomiya M, Acosta PH (2006). Subventricular zone-derived neuroblasts migrate and differentiate into mature neurons in the post-stroke adult striatum. *Journal of Neuroscience*.

[56] Kojima T, Hirota Y, Ema M (2010). Subventricular zone-derived neural progenitor cells migrate along a blood vessel scaffold toward the post-stroke striatum. *Stem Cells*.

[57] Imitola J, Raddassi K, Park KI (2004). Directed migration of neural stem cells to sites of CNS injury by the stromal cell-derived factor 1*α*/CXC chemokine receptor 4 pathway. *Proceedings of the National Academy of Sciences of the United States of America*.

[58] Ohab JJ, Fleming S, Blesch A, Carmichael ST (2006). A neurovascular niche for neurogenesis after stroke. *Journal of Neuroscience*.

[59] Thored P, Arvidsson A, Cacci E (2006). Persistent production of neurons from adult brain stem cells during recovery after stroke. *Stem Cells*.

[60] Yan YP, Sailor KA, Lang BT, Park SW, Vemuganti R, Dempsey RJ (2007). Monocyte chemoattractant protein-1 plays a critical role in neuroblast migration after focal cerebral ischemia. *Journal of Cerebral Blood Flow and Metabolism*.

[61] Bihel E, Pro-Sistiaga P, Letourneur A (2010). Permanent or transient chronic ischemic stroke in the non-human primate: behavioral, neuroimaging, histological, and immunohistochemical investigations. *Journal of Cerebral Blood Flow and Metabolism*.

[62] Burns TC, Verfaillie CM, Low WC (2009). Stem cells for ischemic brain injury: a critical review. *Journal of Comparative Neurology*.

[63] Zhang RL, Zhang ZG, Chopp M (2008). Ischemic stroke and neurogenesis in the subventricular zone. *Neuropharmacology*.

[64] Sugiura S, Kitagawa K, Tanaka S (2005). Adenovirus-mediated gene transfer of heparin-binding epidermal growth factor-like growth factor enhances neurogenesis and angiogenesis after focal cerebral ischemia in rats. *Stroke*.

[65] Wada K, Sugimori H, Bhide PG, Moskowitz MA, Finklestein SP (2003). Effect of basic fibroblast growth factor treatment on brain progenitor cells after permanent focal ischemia in rats. *Stroke*.

[66] Kobayashi T, Ahlenius H, Thored P, Kobayashi R, Kokaia Z, Lindvall O (2006). Intracerebral infusion of glial cell line-derived neurotrophic factor promotes striatal neurogenesis after stroke in adult rats. *Stroke*.

[67] Robin AM, Zhang ZG, Wang L (2006). Stromal cell-derived factor 1*α* mediates neural progenitor cell motility after focal cerebral ischemia. *Journal of Cerebral Blood Flow and Metabolism*.

[68] Cho SR, Benraiss A, Chmielnicki E, Samdani A, Economides A, Goldman SA (2007). Induction of neostriatal neurogenesis slows disease progression in a transgenic murine model of Huntington disease. *Journal of Clinical Investigation*.

[69] Craig CG, Tropepe V, Morshead CM, Reynolds BA, Weiss S, van der Kooy D (1996). In vivo growth factor expansion of endogenous subependymal neural precursor cell populations in the adult mouse brain. *Journal of Neuroscience*.

[70] Kuhn HG, Winkler J, Kempermann G, Thal LJ, Gage FH (1997). Epidermal growth factor and fibroblast growth factor-2 have different effects on neural progenitors in the adult rat brain. *Journal of Neuroscience*.

[71] Androutsellis-Theotokis A, Rueger MA, Park DM (2009). Targeting neural precursors in the adult brain rescues injured dopamine neurons. *Proceedings of the National Academy of Sciences of the United States of America*.

[72] de Chevigny A, Cooper O, Vinuela A (2008). Fate mapping and lineage analyses demonstrate the production of a large number of striatal neuroblasts after transforming growth factor *α* and noggin striatal infusions into the dopamine-depleted striatum. *Stem Cells*.

[73] Fallon J, Reid S, Kinyamu R (2000). In vivo induction of massive proliferation, directed migration, and differentiation of neural cells in the adult mammalian brain. *Proceedings of the National Academy of Sciences of the United States of America*.

[74] Saghatelyan A, de Chevigny A, Schachner M, Lledo PM (2004). Tenascin-R mediates activity-dependent recruitment of neuroblasts in the adult mouse forebrain. *Nature Neuroscience*.

[75] Hargus G, Cui Y, Schmid JS (2008). Tenascin-R promotes neuronal differentiation of embryonic stem cells and recruitment of host-derived neural precursor cells after excitotoxic lesion of the mouse striatum. *Stem Cells*.

[76] Zigova T, Pencea V, Wiegand SJ, Luskin MB (1998). Intraventricular administration of BDNF increases the number of newly generated neurons in the adult olfactory bulb. *Molecular and Cellular Neurosciences*.

[77] Pencea V, Bingaman KD, Wiegand SJ, Luskin MB (2001). Infusion of brain-derived neurotrophic factor into the lateral ventricle of the adult rat leads to new neurons in the parenchyma of the striatum, septum, thalamus, and hypothalamus. *Journal of Neuroscience*.

[78] Huisman E, Uylings HBM, Hoogland PV (2004). A 100% increase of dopaminergic cells in the olfactory bulb may explain hyposmia in parkinson’s disease. *Movement Disorders*.

[79] Winner B, Geyer M, Couillard-Despres S (2006). Striatal deafferentation increases dopaminergic neurogenesis in the adult olfactory bulb. *Experimental Neurology*.

[80] Zhao M, Momma S, Delfani K (2003). Evidence for neurogenesis in the adult mammalian substantia nigra. *Proceedings of the National Academy of Sciences of the United States of America*.

[81] Cooper O, Isacson O (2004). Intrastriatal transforming growth factor *α* delivery to a model of Parkinson's disease induces proliferation and migration of endogenous adult neural progenitor cells without differentiation into dopaminergic neurons. *Journal of Neuroscience*.

[82] Meade CA, Deng YP, Fusco FR (2002). Cellular localization and development of neuronal intranuclear inclusions in striatal and cortical neurons in R6/2 transgenic mice. *Journal of Comparative Neurology*.

[83] Albin RL, Reiner A, Anderson KD, Penney JB, Young AB (1990). Striatal and nigral neuron subpopulations in rigid Huntington’s disease: implications for the functional anatomy of chorea and rigidity-akinesia. *Annals of Neurology*.

[84] Albin RL, Young AB, Penney JB (1990). Abnormalities of striatal projection neurons and N-methyl-D-aspartate receptors in presymptomatic Huntington’s disease. *The New England Journal of Medicine*.

[85] Albin RL, Reiner A, Anderson KD (1992). Preferential loss of striato-external pallidal projection neurons in presymptomatic Huntington’s disease. *Annals of Neurology*.

[86] Kiyama H, Seto-Ohshima A, Emson PC (1990). Calbindin D(28K) as a marker for the degeneration of the striatonigral pathway in Huntington’s disease. *Brain Research*.

[87] Richfield EK, Maguire-Zeiss KA, Vonkeman HE, Voorn P (1995). Preferential loss of preproenkephalin versus preprotachykinin neurons from the striatum of Huntington’s disease patients. *Annals of Neurology*.

[88] Parent JM, Vexler ZS, Gong C, Derugin N, Ferriero DM (2002). Rat forebrain neurogenesis and striatal neuron replacement after focal stroke. *Annals of Neurology*.

[89] Liu F, You Y, Li X (2009). Brain injury does not alter the intrinsic differentiation potential of adult neuroblasts. *Journal of Neuroscience*.

[90] Gustafsson E, Andsberg G, Darsalia V (2003). Anterograde delivery of brain-derived neurotrophic factor to striatum via nigral transduction of recombinant adeno-associated virus increases neuronal death but promotes neurogenic response following stroke. *European Journal of Neuroscience*.

[91] Teramoto T, Qiu J, Plumier JC, Moskowitz MA (2003). EGF amplifies the replacement of parvalbumin-expressing striatal interneurons after ischemia. *Journal of Clinical Investigation*.

